# Design, Modeling, and Fabrication of a High-Q AlN Annular Gyroscope with Sub-10°/h Bias Instability

**DOI:** 10.3390/mi17020268

**Published:** 2026-02-20

**Authors:** Zhenxiang Qi, Jie Gu, Bingchen Zhu, Zhaoyang Zhai, Xiaorui Bie, Wuhao Yang, Xudong Zou

**Affiliations:** 1 State Key Laboratory of Transducer Technology, Aerospace Information Research Institute, Chinese Academy of Sciences, Beijing 100190, China; qizhenxiang21@mails.ucas.ac.cn (Z.Q.); gujie20@mails.ucas.ac.cn (J.G.); zhubingchen22@mails.ucas.ac.cn (B.Z.); biexr@aircas.ac.cn (X.B.); yangwh@aircas.ac.cn (W.Y.); 2 School of Electronic, Electrical and Communication Engineering, University of Chinese Academy of Sciences, Beijing 100049, China; 3 School of Electronics and Integrated Circuits, Aerospace Information Technology University, Jinan 250200, China; zhaizhaoyang20@mails.ucas.ac.cn

**Keywords:** Piezo-MEMS, gyroscope, High-Q factor resonator

## Abstract

This work presents a high-performance piezoelectric MEMS yaw gyroscope fabricated on a single-crystal silicon platform, which achieves a quality factor of 75 k—the highest reported to date among silicon-based piezoelectric gyroscopes. The device employs a wide annular resonator that operates at 132 kHz in the in-plane wineglass mode. To maximize transduction efficiency, we develop an analytical model that relates output charge to the area-integrated in-plane stress under modal deformation, and we use this model to guide parametric optimization of the annular width. The resulting geometry simultaneously enhances the mechanical quality factor and the piezoelectric coupling. A back-etching fabrication process is used to eliminate front-side release holes, thereby preserving structural continuity and suppressing thermoelastic damping. In open-loop rate mode operation with a native frequency split of 28 Hz, the gyroscope demonstrates an angle random walk of 0.34°/√h and a bias instability of 8.19°/h. These performance metrics are comparable to those of state-of-the-art lead zirconate titanate (PZT)-based annular gyroscopes, while the use of lead-free aluminum nitride as the transduction material ensures compliance with RoHS environmental regulations.

## 1. Introduction

Microelectromechanical systems (MEMS) gyroscopes have become pivotal in enabling precision motion sensing across automotive navigation, industrial automation, and consumer electronics, driven by their miniaturization, low power consumption, and scalability [1,2]. Among various transduction mechanisms, electrostatic gyroscopes have long set the performance benchmark [3,4], with capabilities approaching navigation-grade accuracy [5]. By operating in mode-matched resonance [6] and leveraging high-quality (Q) factors [7,8] under vacuum packaging, these devices achieve high scale factor sensitivity and low noise floors. However, their performance is inherently constrained by the nonlinear electrostatic force–displacement relationship in parallel-plate actuators. The pull-in instability limits the maximum allowable drive amplitude, imposing a fundamental trade-off between transduction gain (favored by narrow gaps) and dynamic range (reduced by small gaps). Moreover, electrostatic actuation requires a DC bias voltage, and higher bias voltages yield greater drive efficiency, which increases the complexity and design challenge of the interface circuitry [9,10]. These limitations highlight a fundamental challenge in electrostatic transduction: its reliance on precise, sub-micron gaps and high-voltage biasing not only complicates fabrication and integration but also compromises robustness under real-world mechanical disturbances.

Piezoelectric gyroscopes offer a compelling alternative, combining high electromechanical coupling efficiency, a wide linear actuation range [11], and the elimination of DC polarization voltage required in capacitive designs. Commercial devices like the Silicon Sensing CRM100 [12] achieve sub-10°/h bias instability in open-loop rate mode, enabling a simple interface circuit. This performance is largely attributable to the strong piezoelectric activity of its lead-based PZT, which, however, raises environmental and regulatory concerns.

Aluminum nitride (AlN) has emerged as a promising candidate due to its RoHS compliance and CMOS compatibility. However, its intrinsic piezoelectric coefficient ($d_{31}\approx -2.0$ pC/N) is over two orders of magnitude lower than that of PZT, posing a fundamental challenge for achieving competitive sensitivity. To compensate for this material limitation, careful co-design of the resonator geometry and vibration mode is essential to maximize strain utilization. Since piezoelectric transduction scales with the spatial integral of in-plane stress over the electrode area [13], modes with non-uniform strain, sign reversals, or out-of-plane components inherently limit charge generation, regardless of material quality. Thus, for lead-free platforms, structural integrity and strain-efficient modal design become critical enablers of high sensitivity.

Despite the promise of AlN, demonstrated devices have yet to fully exploit its potential for high-performance gyroscope. The ring gyroscope from [14] shows resonance promise of n = 3 bending mode, but lacks reported gyro performance. The Lissajous frequency-modulated gyroscope from [15] achieves good stability but operates with high ARW and requires complex interface circuit control loops. Moreover, due to its four-mass architecture, the piezoelectric transduction region is confined to the narrow beams between the proof masses, which limits transduction efficiency. The Georgia Tech design [16,17,18], which operates in a high-frequency mode, offers improved motional resistance and bandwidth; however, achieving bias instability below 10°/h necessitates laser trimming combined with eigenmode operation. Moreover, existing TPoS gyroscopes typically exhibit Q-factors below 20 k, a value significantly lower than that of electrostatic counterparts, leading to elevated noise floors and limited resolution. In addition, many of these architectures employ closed-loop control [15] or intricate modulation schemes [17], which compromise the inherent simplicity of piezoelectric transduction.

In this work, we present a lead-free AlN annular gyroscope that addresses these limitations through a holistic co-design of fabrication process and resonator geometry. By eliminating front-side release holes via a back-etching approach, we preserve structural integrity and suppress thermoelastic damping (TED). The annular width is then systematically optimized to simultaneously maximize the mechanical quality factor and piezoelectric transduction efficiency. This co-optimized design enables low-noise, open-loop operation with a simple interface circuit, achieving performance comparable to state-of-the-art PZT-based devices while adhering to environmental regulations.

## 2. Operating Principle

### 2.1. Device Architecture and Mode of Operation

**Figure 1:**
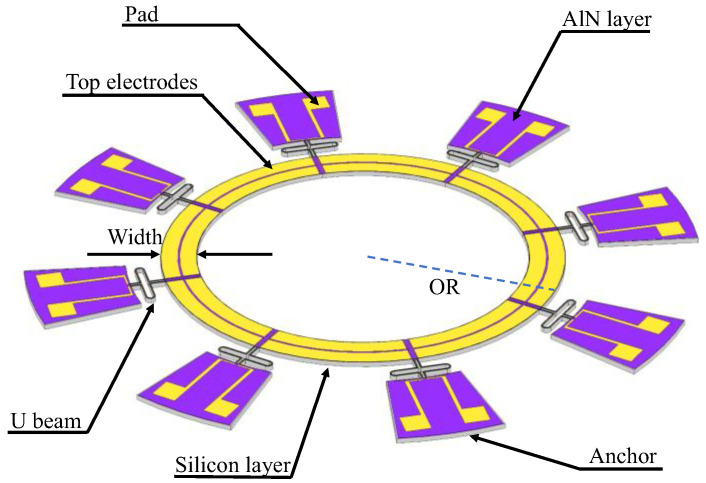
Schematic of the proposed piezoelectric annular MEMS gyroscope. The resonator features a wide annular structure with outer radius (OR) and width, suspended by eight pairs of folded U-shaped beams anchored to the substrate periphery.

Figure 1 illustrates the proposed piezoelectric MEMS gyroscope, which consists of a wide annular resonator supported by eight pairs of folded U beams connected to peripheral anchors.

**Figure 2:**
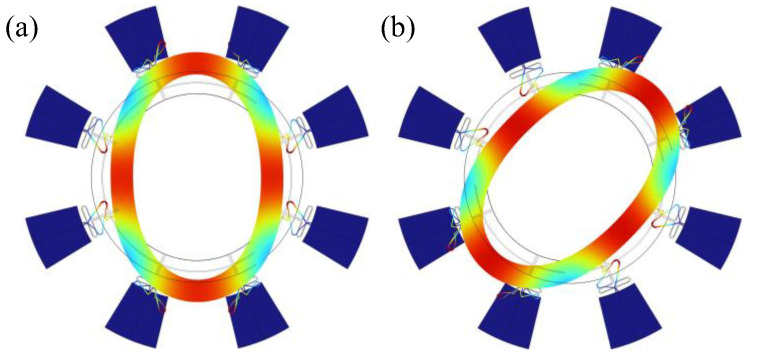
N = 2 wineglass degenerate modes. (**a**) Drive mode, (**b**) sense mode.

The gyroscope operates on a degenerate pair of in-plane second-order wineglass modes enabled by the axisymmetric annular geometry, as illustrated in Figure 2. In operation, one mode is designated as the drive (X) mode and is excited to a constant amplitude, while the orthogonal mode serves as the sense (Y) mode. When an input angular rate $\Omega_{z}$ is applied about the yaw axis, the Coriolis force couples energy from the drive mode to the sense mode, producing a rate-proportional response at the same carrier frequency.

For a rate gyroscope, assume the drive mode amplitude to be $x_{d}$, the mechanical sensitivity of a rate gyroscope can be written as:

(1) $$S_{mech}=\frac{2\lambda x_{d}}{\sqrt{(\frac{{\omega_{s}}^{2}}{\omega_{d}}-\omega_{d}{)}^{2}+\frac{{\omega_{s}}^{2}}{{Q_{s}}^{2}}}}$$

where $Q_{s}$ is the quality factor of sense mode, $\omega_{s}$, $\omega_{d}$ are the angular frequencies of the sense and drive mode. λ represents the Coriolis coupling coefficient.

The angular random walk (ARW) of the gyroscope is then expressed as:

(2) $$MNE\Omega =\frac{1}{2\lambda x_{d}}\sqrt{\frac{4k_{B}T}{\omega_{0}M_{S}Q_{S}}}$$

Collectively, Equations (1) and (2) indicate that the rate output increases with the drive displacement $x_{d}$ and that the sensitivity is maximized when the frequency split between the drive and sense modes is minimized, since a smaller detuning $|\omega_{s}-\omega_{d}|$ reduces the denominator in Equation (1) and thereby enhances Coriolis transduction. It is precisely because high performance requires both large drive amplitudes and near-degenerate mode pairs that we adopt an axisymmetric annular resonator, which inherently supports degenerate wineglass modes with ultra-low frequency splitting, and piezoelectric actuation, which enables large-displacement excitation with high electromechanical efficiency. Moreover, a higher quality factor $Q_{s}$ enhances the resonant gain of the coupled drive–sense dynamics, which not only boosts the rate sensitivity but also suppresses the thermomechanical noise floor, as the noise contribution decreases inversely with $Q_{s}$, leading to a lower ARW.

In practice, the total quality factor $Q_{\mathrm{total}}$ is limited by multiple dissipation mechanisms acting in parallel. According to the reciprocal-addition rule for quality factors, the overall damping is the sum of individual loss contributions:

(3) $$\frac{1}{Q_{\mathrm{total}}}=\frac{1}{Q_{\mathrm{TED}}}+\frac{1}{Q_{\mathrm{anchor}}}+\frac{1}{Q_{\mathrm{surface}}}+\ldots$$

Among these, thermoelastic damping (TED) is typically the dominant intrinsic loss mechanism in high-vacuum environments where air (squeeze-film) damping is negligible. For a vibrating beam or annular resonator, the classic TED-limited quality factor is given by Zener’s formula (as extended by Lifshitz and Roukes):

(4) $$\frac{1}{Q_{\mathrm{TED}}}=\frac{E\alpha^{2}T_{0}}{C_{v}}\cdot \frac{\omega_{0}\tau }{1+(\omega_{0}\tau {)}^{2}}$$

where E is Young’s modulus, α is the coefficient of thermal expansion, $C_{v}$ is the specific heat per unit volume, $T_{0}$ is the ambient temperature, and τ is the thermal relaxation time. Because TED arises from irreversible heat flow during cyclic deformation, it cannot be eliminated by vacuum packaging and often sets the fundamental upper bound on Q for wineglass mode resonators. Critically, both the modal frequency $\omega_{0}$ and the thermal relaxation time τ—and thus $Q_{\mathrm{TED}}$—are strongly dependent on geometric features such as annular width and the presence of etch-release holes. This geometric sensitivity implies that structural design plays a decisive role in minimizing TED, thereby providing the theoretical basis for the geometry-driven optimization pursued in this work.

### 2.2. Piezoelectric Transduction Mechanism

To evaluate the piezoelectric transduction efficiency in the annular resonator, it is essential to quantify the effective mechanical stress experienced by the electrode region under dynamic deformation. Unlike capacitive transducers that rely on gap variation, the charge generation in piezoelectric devices is governed by the integral of stress over the active electrode area. This integral directly determines the open-circuit voltage and, consequently, the signal-to-noise ratio of the sensor.

In piezoelectric transduction, the electric displacement $D_{3}$ generated in a film poled along the thickness direction is related to the in-plane stress σ via the transverse piezoelectric coefficient $d_{31}$:

(5) $$D_{3}=d_{31}\;\sigma$$

The total charge $q_{c}$ collected by an electrode is then the average $D_{3}$ over the active area S:

(6) $$q_{c}=d_{31}\;\sigma_{\mathrm{avg}}\;S$$

Thus, maximizing $q_{c}$ requires not only a large stress magnitude but also a uniform stress sign across the electrode—otherwise, positive and negative contributions cancel.

In our device, we consider an annular beam undergoing in-plane flexural vibration, modeled using the Euler-Bernoulli beam theory. The stress distribution along the electrode segment is derived as a function of azimuthal angle and radial position, enabling the computation of an area-weighted average stress, which serves as a key figure of merit for design optimization.

Within this framework, the bending stress at any point within the beam cross-section is governed by the classical flexure formula:

(7) $$\sigma_{\zeta ,\theta }=-\frac{M\zeta }{I}$$

where M denotes the bending moment, ζ is the distance from the neutral axis, and I is the area moment of inertia.

The bending moment M is related to the radius of the beam. For a circular annular plate of radius R, the moment-curvature relationship can be expressed as [19]:

(8) $$M=\frac{EI}{R^{2}}\frac{\partial }{\partial \theta }\left(v+\frac{\partial^{2}v}{\partial v^{2}}\right)$$

where v represents the radial displacement.

To proceed, the vibration mode shape must be defined. Considering the resonator operating in the n=2 wineglass mode, the radial displacement v is assumed to be:

(9) $$v=\frac{u_{0}}{n}sin(n\theta )$$

where $u_{0}$ is the amplitude of the maximum radial displacement. Substituting Equation (9) into Equation (8) and evaluating for n=2, the bending moment is derived as:

(10) $$M=-\frac{3u_{0}EI}{R^{2}}cos(2\theta )$$

Substituting this expression for M back into the stress formula (Equation (7)) yields the stress distribution along the electrode:

(11) $$\sigma_{\zeta ,\theta }=\frac{3u_{0}E\zeta }{R^{2}}cos(2\theta )$$

Equation (11) reveals two critical characteristics of the stress distribution in the wineglass mode. First, the linear dependence on the local coordinate ζ(σ∝ζ) results in a radial polarity reversal: the inner edge of the annulus (ζ<0) experiences compressive stress while the outer edge (ζ>0) undergoes tensile stress (or vice versa, depending on the phase). Second, the azimuthal variation governed by cos(2θ) produces four alternating tensile and compressive lobes per resonant mode. Crucially, the degenerate drive and sense modes exhibit a 45° spatial phase shift in their respective stress patterns.

To prevent charge cancelation, each electrode must be confined to a region where $\sigma_{\zeta ,\theta }$ maintains uniform sign. This leads to electrodes covering only the inner or outer half and each electrode spans ≤45° to align with one stress lobe.

**Figure 3:**
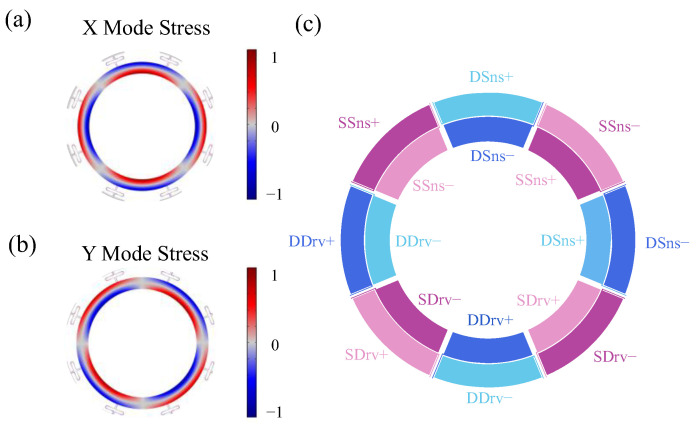
Stress pattern of (**a**) drive mode and (**b**) sense mode. (**c**) The piezoelectric electrodes pattern.

The stress distributions of the drive and sense wineglass modes are shown in Figure 3a,b. Accordingly, we adopted 16 sector-shaped electrodes (Figure 3c), each covering 45° azimuthally and one radial half.

With the electrode geometry defined, integrating the stress over the electrode area, the average stress within the electrode coverage can be obtained:

(12) $$\sigma_{avg}S=\int_{-\frac{\pi }{8}}^{\frac{\pi }{8}}\int_{0}^{\frac{W}{2}}\sigma_{\zeta ,\theta }\left(R+\zeta \right)d\zeta d\theta =\frac{\sqrt{2}u_{0}EW^{2}(3R+W)}{16R^{2}}$$

For completeness, the corresponding electrode area S, with a radial width of W, is given by:

(13) $$S=\frac{\pi W}{32}(4R+W)$$

Combining Equations (12) and (13), the area-averaged stress $\sigma_{\mathrm{avg}}$ is obtained:

(14) $$\sigma_{avg}=\frac{2\sqrt{2}EW(3R+W)}{\pi R^{2}(4R+W)}u_{0}$$

Finally, substituting Equations (13) and (14) into Equation (6):

(15) $$q_{c}=\frac{\sqrt{2}}{16}\;d_{31}\;E\;\;\frac{W^{2}(3R+W)}{R^{2}}u_{0}$$

This expression reveals that $q_{c}$ scales with the square of the electrode width W. A larger W increases both the effective piezoelectric transduction area and the integrated stress over that area, thereby enhancing the total charge output. This implies that a wider annular ring can generate more polarization charge under the same mechanical displacement, which serves as the core theoretical basis for the transduction efficiency optimization presented in Section 3.

Building on the stress-aligned electrode layout, the 16 sector-shaped electrodes (Figure 3c) are configured into four differential pairs to enable independent actuation and sensing of the degenerate wineglass modes:

(1) Drive electrodes for the Drive-mode (DDrv+ and DDrv−).
(2) Sense electrodes for the Drive-mode response detection (DSns+ and DSns−).
(3) Drive electrodes for the sense-mode (SDrv+ and SDrv−)
(4) Sense electrodes of the sense-mode (SSns+ and SSns−) for the Coriolis-induced response detection.

When operating in a fully open-loop mode (where the sense mode does not need to be actuated), SDrv+ and SDrv− can be connected to SSns− and SSns+, respectively, to maximize charge output.

This symmetric and stress-aligned electrode architecture ensures efficient excitation and high-fidelity sensing.

## 3. Multiphysics Optimization and Simulation

This section presents a comprehensive Multiphysics simulation and optimization study of the annular resonator using the finite element method in COMSOL Multiphysics 6.2, which is guided by the theoretical insight that enhancing the mechanical quality factor and piezoelectric transduction efficiency can substantially improve the gyroscope’s sensitivity and noise performance. Because thermoelastic damping stems from irreversible heat flow during vibration and is highly sensitive to strain distribution and thermal boundary conditions, we first examine how a hole-free structural topology reduces strain gradients and improves thermal confinement so that the quality factor is effectively increased. Since the transduction efficiency scales with the integrated polarization charge over the electrode area, which in turn depends on the annular width, we then perform a parametric sweep of the ring width to identify the geometric configuration in which both the quality factor and transduction efficiency are simultaneously maximized. All simulations employ fully coupled electro-thermo-mechanical physics, with boundary conditions detailed in Appendix A. Finally, based on the co-optimization outcome, a specific set of structural dimensions is selected for device implementation so that the fabricated gyroscope achieves high-Q and strong signal transduction within a unified design framework.

### 3.1. Enhancement Mechanism of Hole-Free Structure

To investigate the impact of structural integrity on resonator performance, we perform finite-element simulations focusing on two critical aspects: (1) the mechanical quality factor limited by thermoelastic damping (TED), and (2) the effectiveness of piezoelectric transduction. Unlike electrostatic transduction—which is largely insensitive to local stress distribution—piezoelectric coupling depends directly on the magnitude and uniformity of strain (and thus stress) in the active layer, as reflected in the charge output model (Equation (15)). Therefore, preserving a continuous, hole-free top silicon layer is essential not only for achieving high $Q_{\mathrm{TED}}$ but also for maximizing piezoelectric sensitivity.

It should be noted that our finite-element model employs a key simplification to manage computational complexity. A full simulation including the perforated structure and the piezoelectric layer would introduce an excessive number of degrees of freedom, making the analysis computationally prohibitive. Consequently, the piezoelectric layer was omitted from the current simulation. However, this simplification is well-justified for the purpose of analyzing in-plane vibrational modes. In such modes, the piezoelectric layer and the underlying silicon layer experience identical in-plane strain. Given that stress (σ) is related to strain (ϵ) through the material’s Young’s modulus (E) as σ=Eϵ, the stress in the actual piezoelectric layer can be directly inferred from the simulated silicon stress. Specifically, the two stresses are related by a simple scaling factor equal to the ratio of their respective Young’s moduli, $\sigma_{\mathrm{piezo}}=\sigma_{\mathrm{Si}}\cdot (E_{\mathrm{piezo}}/E_{\mathrm{Si}})$ [19]. This approach allows us to accurately capture the strain distribution, which is the primary driver for piezoelectric transduction, while significantly reducing computational time.

**Figure 4:**
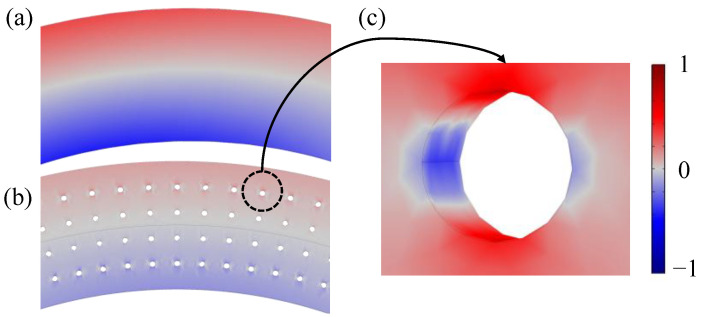
Simulated bending stress in annular resonators. (**a**) Intact annular: symmetric tensile (red) and compressive (blue) stress about the neutral axis (gray). (**b**) Annular with 10 μm radius release holes; stress sign reversal (red in compressive zones) appears near holes. (**c**) Local magnified view near a hole, showing distinct blue regions within the red area, indicating a reduction in average stress.

Figure 4 compares the bending-induced stress distributions in two annular resonator designs. In Figure 4a, the fully intact annular exhibits a classic symmetric stress profile: compressive stress (blue) on one side of the neutral axis (shown in gray) and tensile stress (red) on the other, as expected for pure flexural deformation. In contrast, Figure 4b shows the stress field in an annular with front-side release holes. Notably, within regions that should be under uniform compression (red), the presence of holes disrupts the stress continuity—local areas turn gray or even blue (Figure 4c), indicating a significant reduction in stress and, critically, local sign reversal due to geometric discontinuity. Such anomalous stress patterns degrade both $Q_{TED}$ and the net piezoelectric response, since opposing strains cancel each other in the transduction layer.

**Figure 5:**
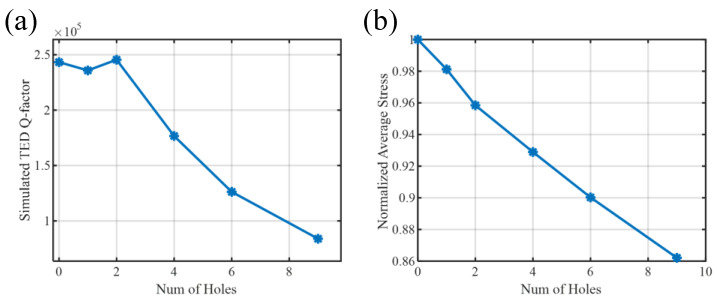
(**a**) Simulated $Q_{\mathrm{TED}}$ and (**b**) normalized surface-averaged stress versus number of release holes (0–9). Holes have 10 μm radius and 1.125° circumferential spacing. Both metrics degrade with increasing hole count.

This trend is quantified in Figure 5a, which plots the simulated TED-limited quality factor ($Q_{\mathrm{TED}}$) and the surface-averaged stress magnitude as a function of the number of radial release holes (from 0 to 9). All holes are modeled with a fixed radius of 10 μm and equally spaced at 1.125° angular intervals along the annular circumference. As the number of holes increases, $Q_{\mathrm{TED}}$ decreases monotonically—from 250,000 (hole-free) to 84,000 with nine holes—due to enhanced localized dissipation from stress perturbations induced by the geometric discontinuities. Concurrently, the surface-averaged stress drops from its maximum value (normalized to 1.0) to 0.86, directly reducing the effective piezoelectric coupling. These results confirm that eliminating release holes (e.g., via back-side etching) preserves both high-Q and strong electromechanical transduction, making it particularly advantageous for piezoelectric MEMS gyroscopes.

### 3.2. Parametric Sweep of Annular Width

To evaluate the influence of structural geometry on resonator performance, we perform a parametric sweep of the annular width w from 160 μm to 760 μm while keeping the outer radius fixed at 2000 μm. The resonator consists of a 50 μm thick <111> silicon device layer (electrically grounded) and a 2 μm thick piezoelectric layer (Aluminum Nitride). The top electrodes are configured according to the terminal layout in Figure 3c.

Across all studies, the core Multiphysics coupling includes solid mechanics and electrostatics, linked via the piezoelectric effect.

#### 3.2.1. Eigenfrequency Study with Thermoelastic Damping

**Figure 6:**
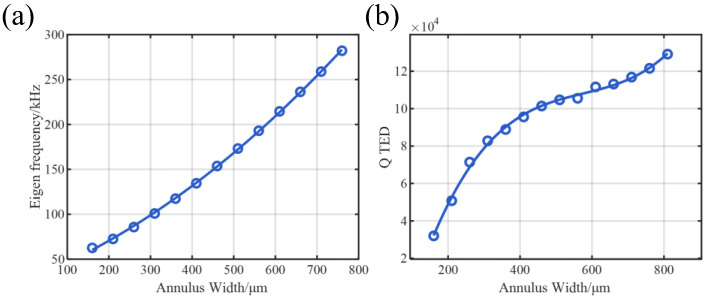
Resonant frequency and TED-limited quality factor versus annular width. (**a**) The eigenfrequency increases monotonically with width due to structural stiffening. (**b**) The growth rate of $Q_{TED}$ exhibits a strong dependence on width: A sharp initial rise is followed by a pronounced plateau region (450–700 μm) of significantly diminished returns, before a recovery of faster growth beyond 700 μm. This plateau indicates an unfavorable trade-off, where large frequency increases yield minimal Q benefit.

To evaluate damping, an eigenfrequency study is performed with thermoelastic damping (TED) enabled. This requires the addition of the heat transfer in solid physics and the thermal expansion Multiphysics coupling to model irreversible heat flow during cyclic deformation. From the complex eigenfrequencies, we extract the fundamental wineglass-mode resonant frequency (Figure 6a) and the TED-limited quality factor $Q_{\mathrm{TED}}$ (Figure 6b).

As depicted in Figure 6a, the eigenfrequency exhibits a monotonic increase with the annulus width, consistent with the expected stiffening of the structure. In contrast, the TED-limited quality factor ($Q_{TED}$) reveals a more complex, non-linear dependence on width. Initially, $Q_{TED}$ rises sharply as the width increases from 160 μm, indicating effective mitigation of thermoelastic dissipation. However, a distinct plateau region emerges between 450 μm and 700 μm, where the rate of $Q_{TED}$ improvement significantly diminishes. This saturation suggests that the structure enters a transition zone where the assumptions of classical curved beam theory begin to break down, and the strain energy distribution changes significantly. Beyond 700 μm, the $Q_{TED}$ recovers a steeper increasing trend, suggesting a transition in the geometric constraints governing the thermoelastic dissipation.

These results provide crucial guidelines for device optimization. Within the plateau region (450–700 μm), the eigenfrequency increases significantly (by approximately 100 kHz), while the $Q_{TED}$ shows negligible improvement. From a design perspective, this represents an unfavorable trade-off: the structural modification effectively shifts the operating point to a higher frequency without yielding a corresponding enhancement in quality factor.

It is advisable to avoid the plateau regime. Instead, an optimal balance is achieved at the terminal end of the initial rapid-rise region (around 350–450 μm), where a high $Q_{TED}$ is attained without incurring the penalty of unnecessary frequency escalation.

#### 3.2.2. Electromechanical Transduction Under Fixed Q

For transduction performance analysis, a frequency-domain harmonic response study is conducted without thermal physics. Mechanical damping is manually introduced to enforce a constant total quality factor of $Q=10^{4}$ across all geometries, thereby isolating the impact of structural geometry on electromechanical coupling. From the simulated electrical response at resonance, we extract the sensing current per unit drive displacement and the motional resistance.

**Figure 7:**
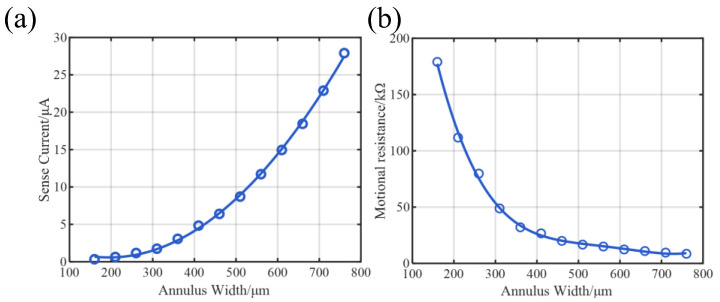
Electromechanical transduction performance under a fixed total Q ($10^{4}$). (**a**) Sense current per unit drive displacement increases with annular width, benefiting from a larger piezoelectric coupling area. (**b**) Motional resistance ($R_{m}$) decreases but shows diminishing returns beyond 400 μm. The combined results suggest that further width increase yields marginal electrical benefits while potentially exacerbating system-level issues like bias instability.

As shown in Figure 7a, the sense current per unit displacement ($I_{\mathrm{sense}}/x_{d}$) increases monotonically with the annulus width, driven by the enlargement of the effective electromechanical coupling area. Concurrently, Figure 7b presents a significant reduction in motional resistance ($R_{m}$), which is desirable for improving SNR. However, the rate of improvement in $R_{m}$ exhibits diminishing returns beyond 400 μm, where the curve flattens considerably, suggesting marginal electrical benefits despite further geometric expansion.

From a system-level perspective, while higher sense current can be achieved at elevated resonant frequencies, this benefit may come at the cost of degraded bias instability. Under fixed stiffness coupling conditions, higher frequencies tend to amplify the drift on bias stability, potentially undermining long-term performance [20]. Thus, considering rising drive power and diminishing efficiency returns, a width of 350–450 μm offers a balanced compromise, ensuring sufficient sense current and robust operation.

### 3.3. Optimal Parameter Determination

As demonstrated in Section 3.1, maintaining a hole-free top silicon layer is critically advantageous. Therefore, the no-hole design is adopted, with geometric optimization focused solely on annular width.

Section 3.2 further reveals that annular width plays a pivotal role in balancing stiffness, resonance frequency, $Q_{\mathrm{TED}}$, and transduction gain.

Taking these trade-offs into account, the optimal design lies in a balanced regime. The width range of 350–450 μm emerges as such a compromise. Within this interval, $Q_{\mathrm{TED}}$ is almost close to its saturated value in a flat area and the motional resistance is sufficiently low.

Accordingly, the final design adopts an annular width of 420 μm, selected near the upper end of the favorable range (350–450 μm) to prioritize transduction gain while avoiding the onset of diminishing returns. When combined with the hole-free architecture, this choice provides a favorable trade-off among mechanical quality factor, resonant frequency, and piezoelectric transduction efficiency. The key geometry and parameters of the final design are summarized in Table 1. This configuration serves as the basis for prototype fabrication and experimental validation in the following sections.

## 4. Fabrication Process

**Figure 8:**
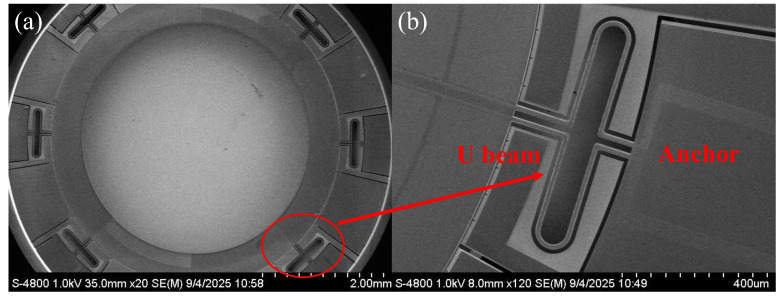
SEM pictures of the presented gyroscope. (**a**) Top-down view of the entire device; (**b**) enlarged view near the folded beams, highlighting the local connection between the resonant annular and the anchor.

SEM pictures of the top-down view of the fabricated gyroscope are shown in Figure 8a. Routing traces on each beam connect the piezoelectric electrodes to bonding pads at the anchor for wire bonding, as shown in Figure 8b.

**Figure 9:**
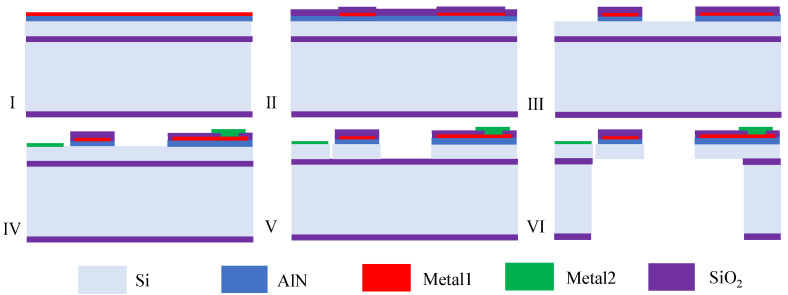
Fab process. (**I**) Sputter deposition of 2 μm AlN directly on highly doped SOI device layer. (**II**) Ti/Au Metal1 patterning by IBE. (**III**) SiO_2_ hard mask deposition and patterning for AlN wet etch in TMAH. (**IV**) Cr/Au Metal2 bonding pads formed by lift-off; (**V**) DRIE of 50 μm device layer to define annular resonator and folded beams. (**VI**) Backside DRIE through 300 μm handle wafer and final RIE removal of residual BOX to fully release the structure.

The fabrication process of the gyroscope is shown in Figure 9. A 2 μm thick AlN film is sputter-deposited directly onto the heavily doped ⟨111⟩ silicon device layer of an SOI wafer, without a dedicated metal bottom electrode. The conductive silicon layer serves as the ground electrode, simplifying fabrication and reducing interfacial losses.

Metal1 (Ti/Au) electrodes are patterned using high-precision ion beam etching (IBE). A 500 nm SiO_2_ layer is deposited by ICP CVD and patterned to act as a hard mask for the subsequent AlN wet etch. The AlN layer is selectively etched in a 25 wt% TMAH solution at 50 °C for approximately 10 min [21,22]. Following AlN patterning, the SiO_2_ layer remains in place to protect and electrically insulate the underlying Metal1, providing robust long-term dielectric isolation. Next, metal2 (Cr/Au) is used to define the bonding pads. The SiO_2_ hard mask is first opened in the pad areas via RIE, followed by deposition of Metal2 and lift off to form low-resistance bonding pads for electrical interconnection. The mechanical structure of the SOI device layer is defined by deep reactive ion etching (DRIE). Using the Bosch process, the 50 μm thick device layer is etched to form the wide annular resonator and its supporting beams. For backside processing, a thick photoresist layer is spin-coated onto the wafer backside. The buried oxide on the back of the handle layer is locally removed by reactive ion etching (RIE) in the release regions to expose the underlying silicon. A subsequent backside DRIE step fully etches through the 300 μm thick silicon handle layer [23,24], creating a trench that releases the movable annular structure.

This backside trench release approach eliminates the need for front-side release holes, which are typically required in HF-based wet etching processes. As a result, the structural integrity and in-plane stress continuity of the piezoelectric resonator are preserved, thereby reducing TED and improving transduction efficiency. Finally, the remaining BOX layer is removed by RIE to fully release the structure.

## 5. Experimental Results

**Figure 10:**
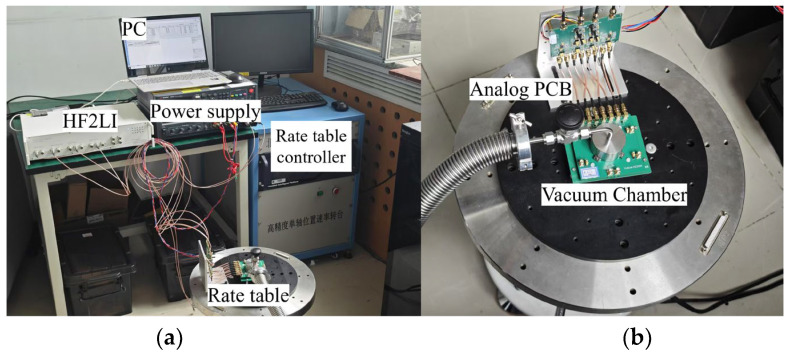
Experimental test setup for gyroscope characterization. (**a**) Overview of the measurement environment, showing the vacuum chamber mounted on a precision rate table, along with the Zurich Instruments HF2LI lock-in amplifier and supporting power supplies. (**b**) Close-up view of the vacuum chamber on the rate table, highlighting the sealed cap enclosing the gyroscope PCB and the SMA coaxial connections to the analog interface board. The system maintains a pressure of ~0.01 Pa to suppress squeeze-film damping.

Experimental measurements were carried out in a custom-designed vacuum chamber equipped with mechanical and molecular pumps. A sealed cap was placed over the printed circuit board (PCB) to enclose the gyroscope, as illustrated in Figure 10.

To reduce air leakage, vacuum grease was applied to the contact area between the vacuum chamber and the PCB. The chamber maintained a vacuum pressure of approximately 0.01 Pa, as monitored by an integrated gauge, effectively minimizing squeeze-film damping (SFD) effects.

The gyroscope was connected via SMA coaxial connectors to an analog interface board that implements transimpedance amplification (TIA), single-ended-to-differential conversion for excitation, and differential-to-single-ended conversion for output sensing. The processed signals were then interfaced to a Zurich Instruments HF2LI lock-in amplifier, which provided the excitation drive and performed demodulated signal readout.

**Figure 11:**
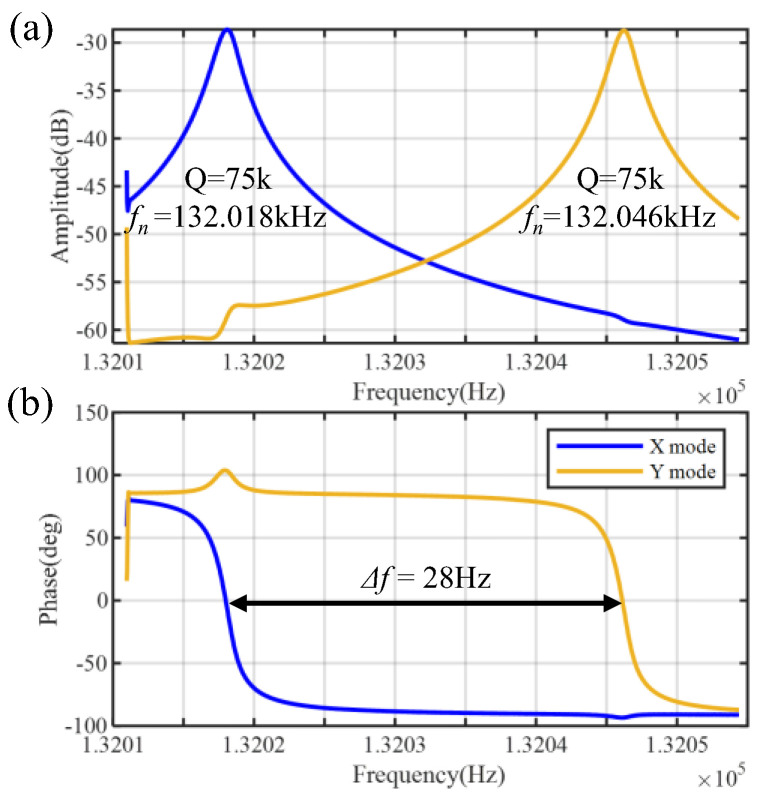
Frequency sweep of (**a**) amplitude and (**b**) phase of the gyroscope under vacuum (~0.01 Pa). The resonant frequency is approximately 132 kHz, with an initial frequency split of 28 Hz and a quality factor (Q) of 75,000.

The resonator performance of the gyroscope is first characterized. In this measurement, the piezoelectric electrodes for the Y-mode are configured exactly as shown in Figure 3c. Frequency sweeps are simultaneously applied to both the X- and Y-modes, yielding the amplitude and phase responses shown in Figure 11a and Figure 11b, respectively. At room temperature and under vacuum pressure, the gyroscope resonant frequency is approximately 132 kHz, with an initial frequency split of about 28 Hz and a Q-factor of 75 k. This value reaches 78% of the simulated thermoelastic damping (TED) limit of 96 k shown in Table 1, indicating that TED is the dominant energy dissipation mechanism.

**Figure 12:**
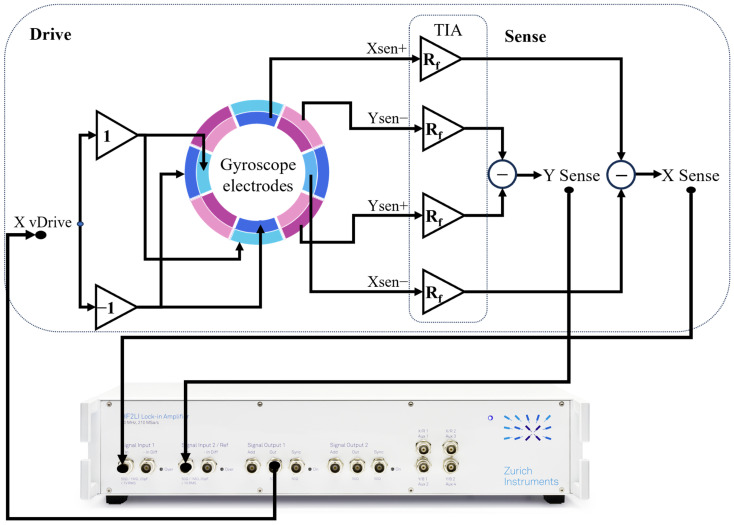
Detailed electrical connection schematic for gyroscope performance testing.

The detailed electrical connection schematic for gyroscope performance testing is shown in Figure 12. The Output 1 of the HF2LI is connected to the X-mode voltage drive input, which—through a single-ended-to-differential converter—drives the piezoelectric excitation electrodes of the X-mode. The differential sensing electrodes for both X- and Y-modes are first amplified by TIAs, then converted to single-ended signals via differential-to-single-ended circuits and finally routed to Input 1 and Input 2 of the lock-in amplifier, respectively.

The HF2LI employs a phase-locked loop (PLL) and automatic gain control (AGC) on Channel 1 to maintain the X-mode oscillating at its resonant frequency with constant amplitude. The Y-mode signal is demodulated at the X-mode’s resonant frequency to extract the angular rate output.

Notably, among the eight electrodes associated with the X-mode, four are dedicated to drive and four to sense. In contrast, the Y-mode operates in a fully open-loop configuration; thus, during gyroscopic operation, all eight Y-mode electrodes are used exclusively for sensing to maximize angular rate sensitivity.

Owing to the high-quality factor (Q) of the gyroscope, the dynamic resistance at resonance is calculated to be approximately 4 kΩ, reflecting strong electromechanical coupling efficiency. The device is driven with a 38 mV differential AC voltage, resulting in a drive-mode displacement amplitude of $x_{d}=0.55\;\mu m$. The device is driven with a 38 mV differential AC voltage, resulting in a drive-mode displacement amplitude of $x_{d}=0.55\;\mu m$. Rate response up to 90°/s is measured at the frequency of 0.25 Hz, where the applicable rotation rate is limited by the testing setup.

**Figure 13:**
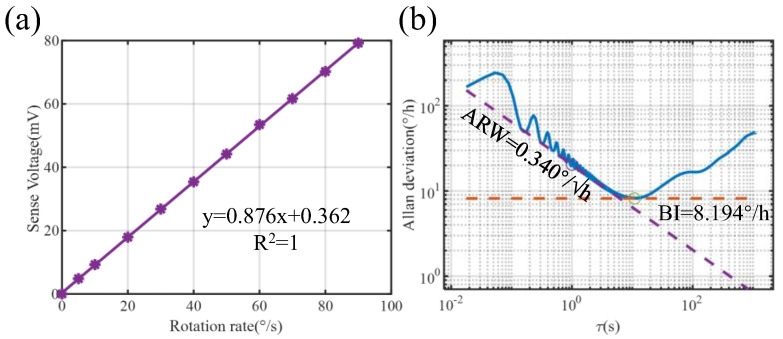
Gyro performance test. (**a**) Sensitivity obtained by measuring the sinusoidal response; (**b**) Allan deviation.

The gyroscope sensitivity, obtained from the in-phase channel of the Y-mode, is presented in Figure 13a. The angular random walk (ARW) and bias instability (BI) are characterized without active temperature control. The Allan deviation plot in Figure 13b shows the ARW and BI of the gyroscope, yielding an ARW of 0.340°/√h and a bias instability of 8.194°/h. Table 2 summarizes a performance comparison with previously reported state-of-the-art piezoelectric MEMS gyroscopes from both academia and industry. As shown in Table 2, the open-loop bias instability (BI) of the proposed AlN gyroscope is comparable to that of PZT-based devices, despite AlN’s significantly lower piezoelectric coefficient. Notably, this performance is achieved using a Pb-free, RoHS-compliant material, offering a sustainable alternative to lead-based piezoelectric gyro.

## 6. Conclusions

This work demonstrates a high-performance MEMS yaw gyroscope based on aluminum nitride (AlN) piezoelectric transduction, fabricated on a ⟨111⟩ single-crystal silicon device layer. Through co-design of the annular resonator geometry, electrode placement that maximizes strain-integrated coupling, and a backside trench release process that eliminates front-side etch holes, structural integrity and in-plane stress continuity are preserved—enabling effective suppression of thermoelastic damping. As a result, the device achieves a quality factor of 75,000, which represents the highest reported value to date for silicon-based piezoelectric gyroscopes. Operating in open-loop rate mode with a native frequency split of 30 Hz, it attains an angle random walk of 0.34°/√h and a bias instability of 8.19°/h, performance metrics that rival those of state-of-the-art PZT-based counterparts. Critically, this performance is achieved using a lead-free, RoHS-compliant AlN transduction layer. These results confirm that, when supported by judicious structural and process co-optimization, AlN can deliver performance parity with lead-based piezoelectrics—offering a sustainable pathway toward high-stability, environmentally compliant inertial sensors.

## Figures and Tables

**Table 1:** Key geometry and parameters of the AlN annular gyroscope.

Parameter	Value
Annular width	420 μm
Silicon device layer thickness	50 μm
Silicon crystal orientation	<111>
Suspension beam width	17 μm
AlN thickness	2 μm
Simulated resonant Frequency	135 kHz
Simulated $Q_{\mathrm{TED}}$	96 k

**Table 2:** Overview of published performance for piezoelectric gyroscopes.

Ref	Year	Piezo Material	Mode	Frequency	Q-factor	BI (°/h)	ARW(°/√h)
[3]	2022	AlN	n = 3 In-plane	457 kHz	16.5 k	--	--
[5]	2023	AlN	Baw	3 MHz	10.6 k	8.6	0.145
[4]	2024	AlN	Tuning fork	67 kHz	~7 k	5	6.66
[2]	--	PZT	n = 2 Wineglass	22 kHz	--	~8	0.2
This work	2026	AlN	n = 2 Wineglass	132 kHz	75 k	8.2	0.34

**Figure A1:**
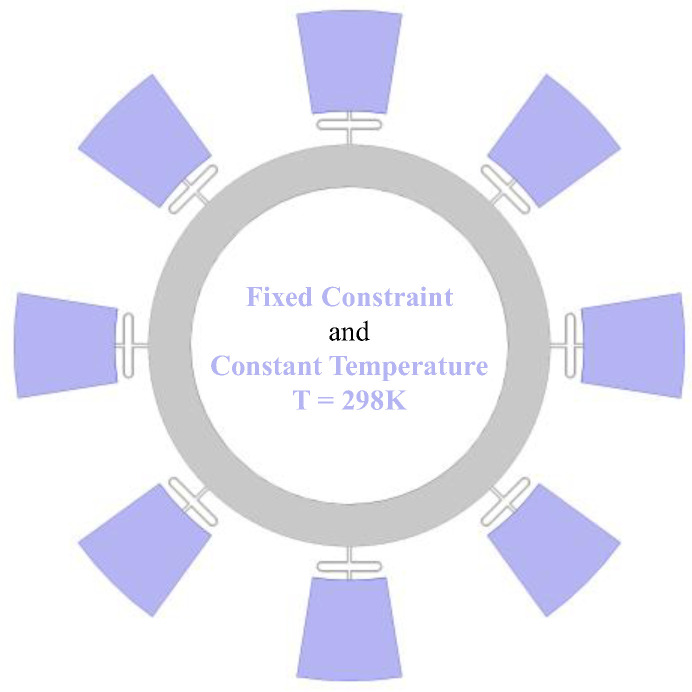
Mechanical fixed constraints and thermal boundary conditions (298 K) applied to the bottom surfaces of the eight anchors.

**Figure A2:**
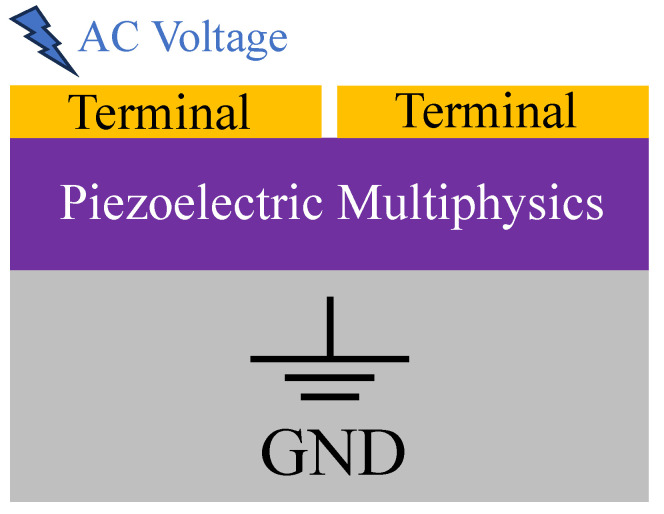
Electrostatic boundary conditions. Owing to the TPoS architecture, the silicon layer is grounded, while the top electrodes on the piezoelectric layer are defined as voltage terminals that are used for actuation and sensing and are coupled to the mechanical domain through the piezoelectric Multiphysics interface. The terminal numbering and corresponding applied potentials are listed in Table A1.

## Data Availability

The original contributions presented in this study are included in the article. Further inquiries can be directed to the corresponding author.

## Appendix A. Multiphysics Setup and Boundary Conditions

The Multiphysics simulations and optimization presented in this work were carried out using COMSOL Multiphysics version 6.2. The model couples three physics interfaces: Solid Mechanics, Electrostatics, and Heat Transfer in Solids, with Multiphysics couplings enabled for Piezoelectric Effect and Thermal Expansion.

As illustrated in Figure 1, in the Solid Mechanics interface, mechanical boundary conditions consist of fixed constraints applied to the bottom surfaces of the eight anchor points, as shown in Figure A1; all other structural regions are left free to deform.

In the Heat Transfer in Solids interface, a constant temperature boundary condition of 298 K (25 °C) is applied to the same anchor bottom surfaces used for mechanical fixation (i.e., the eight anchor bases), as depicted in Figure A1. This configuration approximates thermal anchoring to the underlying package or substrate, which serves as a heat sink during operation.

For the Electrostatics interface, the silicon layer of the resonator is assigned as a bulk terminal grounded at 0 V. The top electrodes on the AlN piezoelectric layer are defined as surface voltage terminals according to the layout in Figure 3c, with the specific voltage assignments for each electrode listed in Table A1.

**Table A1:** Voltage assignments for the top electrode terminals in the electrostatics interface.

Electrodes	Terminal Number	Voltage
DDrv+	1	+10 mV
DDrv−	2	−10 mV
DSns+	3	0 V
DSns−	4	0 V
SSns+/SDrv−	5	0 V
SSns−/SDrv+	6	0 V
